# Micro-ultrasound guided versus multiparametric MRI-guided prostate biopsy: a systematic review and meta-analysis

**DOI:** 10.1007/s11255-026-05227-9

**Published:** 2026-06-15

**Authors:** Niamh Moynagh, Josh Bruinsma, Benjamin M. Mac Curtain, Gavin G. Calpin, Mohamed Elfaki, Rostyslav Nikolenko, Avinash Deshwal, Nicholas Clausen, Ali Benour, Wanyang Qian, Hugo C. Temperley, Peter Lawlor, John Keane, Claudiu Cozman, Lee Chien Yap, Padraig J. Daly

**Affiliations:** 1 Royal College of Surgeons in Ireland, 123 St Stephens Green, Dublin 2, Dublin, Ireland; 2 Department of Urology, University Hospital Waterford, Dunmore Road, Waterford, X91 ER8E Ireland; 3 Department of Urology, Royal Perth Hospital, Perth, WA Australia; 4 Northwell Health, New Hyde Park, Long Island, NY USA; 5 Department of Urology, Lenox Hill Hospital, New York, NY USA; 6 Department of Surgery, Beaumont Hospital, Dublin, Ireland; 7 Department of Surgery, St. John of God Midland Hospital, Perth, WA Australia; 8 Department of Radiology, St. James’s Hospital, Dublin, Ireland

**Keywords:** Microultrasound, Multiparametric MRI, Biopsy, Prostate cancer, Detection rates

## Abstract

**Purpose:**

To investigate patients with suspected prostate cancer, multiparametric MRI (mpMRI) has been the standard imaging modality employed. However, mpMRI is costly and not universally available. Microultrasound (mUS), a novel 29-MHz modality, offers high-resolution, real-time prostate imaging and may guide biopsy with comparable diagnostic accuracy. This systematic review and meta-analysis compares mUS-guided and mpMRI-guided prostate biopsy for the detection of prostate cancer (PCa).

**Methods:**

A systematic search was conducted up to April 2025, in accordance with the PRISMA guidelines (PROSPERO ID CRD420251033768). Comparative studies reporting PCa detection between mUS and mpMRI were included.

**Results:**

Twelve studies comprising 4318 patients (3475 mUS; 3615 mpMRI) were included. There was no significant difference between modalities in the detection of clinically significant prostate cancer (csPCa), both overall (RR 0.97; 95%CI 0.90–1.05) and with targeted biopsy (RR 1.05; 95%CI 0.97–1.14). Overall cancer detection was also comparable (RR 1.00; 95%CI 0.87–1.15), as was Grade Group 1 prostate cancer detection, whether assessed across all biopsies (RR 1.00; 95%CI 0.72–1.39) or with targeted biopsy alone (RR 0.90; 95%CI 0.64–1.27). Weighted pooled values showed mUS had a sensitivity of 93.2%, specificity of 23.3%, positive predictive value (PPV) of 46.4%, and negative predictive value (NPV) of 81.2%, compared with mpMRI’s sensitivity of 87.5%, specificity of 25.7%, PPV of 44.6%, and NPV of 73.7%.

**Conclusion:**

mUS-guided biopsy demonstrates diagnostic performance comparable to mpMRI-guided biopsy for PCa detection and represents a promising alternative or adjunct to mpMRI in prostate cancer diagnostic pathways.

## Introduction

Prostate cancer remains one of the most diagnosed malignancies worldwide, and its early detection is crucial for appropriate risk stratification, treatment planning and prognostication [1]. Traditionally, prostate cancer diagnosis relied on systematic transrectal ultrasound (TRUS)-guided biopsies. However, the advent of multiparametric magnetic resonance imaging (mpMRI) has transformed the diagnostic pathway by improving the detection of clinically significant prostate cancer (csPCa) while minimizing overdiagnosis of indolent disease [2].

Despite its diagnostic advantages, mpMRI is associated with several limitations, including high cost, limited accessibility, requirement for specialised radiological expertise, and contraindications such as the presence of ferromagnetic implants or impaired renal function [3–5]. Moreover, variability in image interpretation and patient-related factors can reduce mpMRI’s diagnostic consistency across centres [4].

High-resolution microultrasound (mUS) has recently emerged as a promising alternative imaging modality. Operating at 29 MHz, mUS offers real-time imaging with a spatial resolution down to 70 microns, enabling direct visualisation of prostate tissue architecture [6, 7]. mUS is scored using the Prostate Risk Identification using Microultrasound (PRI-MUS) system, analogous to the PI-RADS system used for mpMRI [6].

Several comparative studies have suggested that mUS achieves diagnostic performance comparable to that of mpMRI for guiding biopsies in detecting csPCa which may result from the clinician’s ability to perform real-time targeting during biopsy [8–12]. While individual studies have compared mUS and mpMRI across various clinical settings including biopsy-naïve populations, active surveillance cohorts, and men undergoing confirmatory biopsies, a comprehensive synthesis of the evidence is lacking [8, 12, 13]. Some trials have evaluated diagnostic sensitivity and specificity, while others have focused on clinical utility in avoiding unnecessary biopsies or mpMRI usage [8, 13, 14].

This systematic review and meta-analysis aims to compare the diagnostic accuracy, sensitivity, specificity, and clinical utility of mUS biopsy versus mpMRI-guided biopsy for the detection of clinically significant prostate cancer. By synthesising data from available comparative studies, we seek to clarify the role of mUS as a potential standalone imaging modality or adjunct to current diagnostic pathways.

## Methods

This systematic review was performed in accordance with the Preferred Reporting Items for Systematic Reviews and Meta-Analyses (PRISMA) guidelines [15]. Authors contributed to formulating the study protocol and it was then registered with the International Prospective Register of Systematic Reviews (PROSPERO) (registration ID CRD420251033768).

### Patient, intervention, comparison, outcome

Using the PICO framework [16], the aspects the authors wished to address were:

*Population* – Patients with a clinical suspicion of prostate cancer undergoing primary biopsy and patients on an active surveillance protocol for prostate cancer undergoing re-biopsy.

*Intervention* – mUS-guided prostate biopsy.

*Comparison* – mpMRI-guided prostate biopsy.

*Outcomes–Detection* rates of csPCa defined as ISUP grade group 2 disease or greater.

### Search strategy

An electronic search was performed of the *PubMed, Embase,* and *Central* databases for relevant studies. This search was performed by three independent reviewers (JB, RN, & AB). This search included the search terms: (micro ultrasound), (micro US), (microultrasonography), (MRI), (magnetic resonance imaging), and (prostate biopsy) with ‘AND’ and ‘OR’ used as Boolean operators. Included studies were limited to the English language. The search was not restricted by year of publication. All duplicate studies were manually removed, before titles were screened, and studies considered appropriate had their abstracts and/or full text reviewed. In cases of several manuscripts using the same patient dataset, the most relevant dataset was included in the analysis. In cases of discrepancies, the study was included for full text review, and a fourth author reviewed all manuscripts (NM). The reference lists of included studies were also screened for additional relevant studies. The final search was performed on the 17th April 2025.

### Inclusion criteria

(1) Studies reporting outcomes of mUS-guided and mpMRI-guided prostate biopsy.

(2) Detection rates of PCa reported.

(3) Original research studies (prospective and retrospective).

### Exclusion criteria

(1) Comparing PCa detection rates of targeted biopsy versus systematic biopsy.

(2) Only the abstract is available for review.

(3) Published abstracts from conference proceedings, review articles, case reports, and editorial articles.

(4) Animal or cadaveric studies.

(5) Not meeting the inclusion criteria.

### Data extraction

The following data were extracted and collated from retrieved studies meeting inclusion criteria: (1) First author name, (2) year of publication, (3) country of origin, (4) study type, (5) journal, (6) number of patients, (7) outcomes of interest.

### Statistical analysis and quality assessment

Data were expressed as dichotomous outcomes, reported as risk ratios (RRs) expressed with 95% confidence intervals (CIs) following estimation using the Mantel–Haenszel method. Statistical heterogeneity was determined using *I*^*2*^ statistics. Fixed or random-effects models were applied based on whether significant heterogeneity (*I*^*2*^ > 50%) existed between studies included in the analysis. All tests of significance were two-tailed with *P* < 0.05 indicating statistical significance. Meta-analysis was performed using Review Manager (RevMan), Version 5.4 (Nordic Cochrane Centre, Copenhagen, Denmark) [17]. All studies with a comparator group were included in the meta-analysis whilst studies lacking a comparator were included in the narrative quantitative analysis only. Risk of bias and methodology quality assessment was performed in accordance with ROB 2 and Modified Newcastle–Ottawa scale. Risk of bias for the single randomized controlled trial (RCT) was assessed using the Cochrane RoB 2 tool, evaluating the five standard domains: randomization process, deviations from intended interventions, missing outcome data, measurement of the outcome, and selection of the reported result. Each domain was rated low risk, some concerns, or high risk and summarized with an overall judgment.

For all non-randomized comparative diagnostic studies, we applied the ROBINSI (Risk Of Bias In Nonrandomized Studies of Interventions) tool, covering confounding, selection of participants, classification of interventions, deviations from intended interventions, missing data, measurement of outcomes, and selection of reported results. Each domain was graded as low, moderate, serious, or critical risk of bias, with an overall judgment assigned according to the ROBINSI guidance and Rob2 Assessment for RCTs, with results tabulated in Tables 1, 2 and 3.

**Table 1:** RoB 2 Assessment of RCT

Study	Randomization process	Deviations from interventions	Missing data	Measurement of outcome	Selection of reported result	Overall
Kinnaird 2025	Low	Low	Low	Low	Some concerns	Low

**Table 2:** Nonrandomised studies ROBISNI summary

Study	Confounding	Selection	Classification	Deviations	Missing data	Measurement	Reporting	Overall
Albers 2022	Low	Moderate	Serious	Low	Low	Some concerns	Moderate	Moderate
Beatrici 2024	Moderate	Serious	Low	Low	Low	Some concerns	Moderate	Moderate
Avolio 2024	Moderate	Moderate	Low	Low	Low	Low	Moderate	Moderate
Ghai 2022	Low	Moderate	Low	Low	Low	Some concerns	Low	Moderate
Hofbauer 2022	Low	Moderate	Low	Low	Low	Some concerns	Moderate	Moderate
Klotz 2021	Moderate	Moderate	Serious	Low	Low	Some concerns	Moderate	Moderate
Lorusso 2022	Serious	Serious	Moderate	Low	Low	Low	Moderate	Moderate
Lughezzani 2021	Moderate	Moderate	Low	Low	Low	Some concerns	Moderate	Moderate
Maffei 2023	Moderate	Moderate	Moderate	Low	Low	Some concerns	Moderate	Moderate
Piccolini 2024	Moderate	Moderate	Low	Low	Low	Some concerns	Moderate	Moderate
Wiemer 2021	Low	Moderate	Low	Low	Low	Some concerns	Moderate	Moderate

**Table 3:**
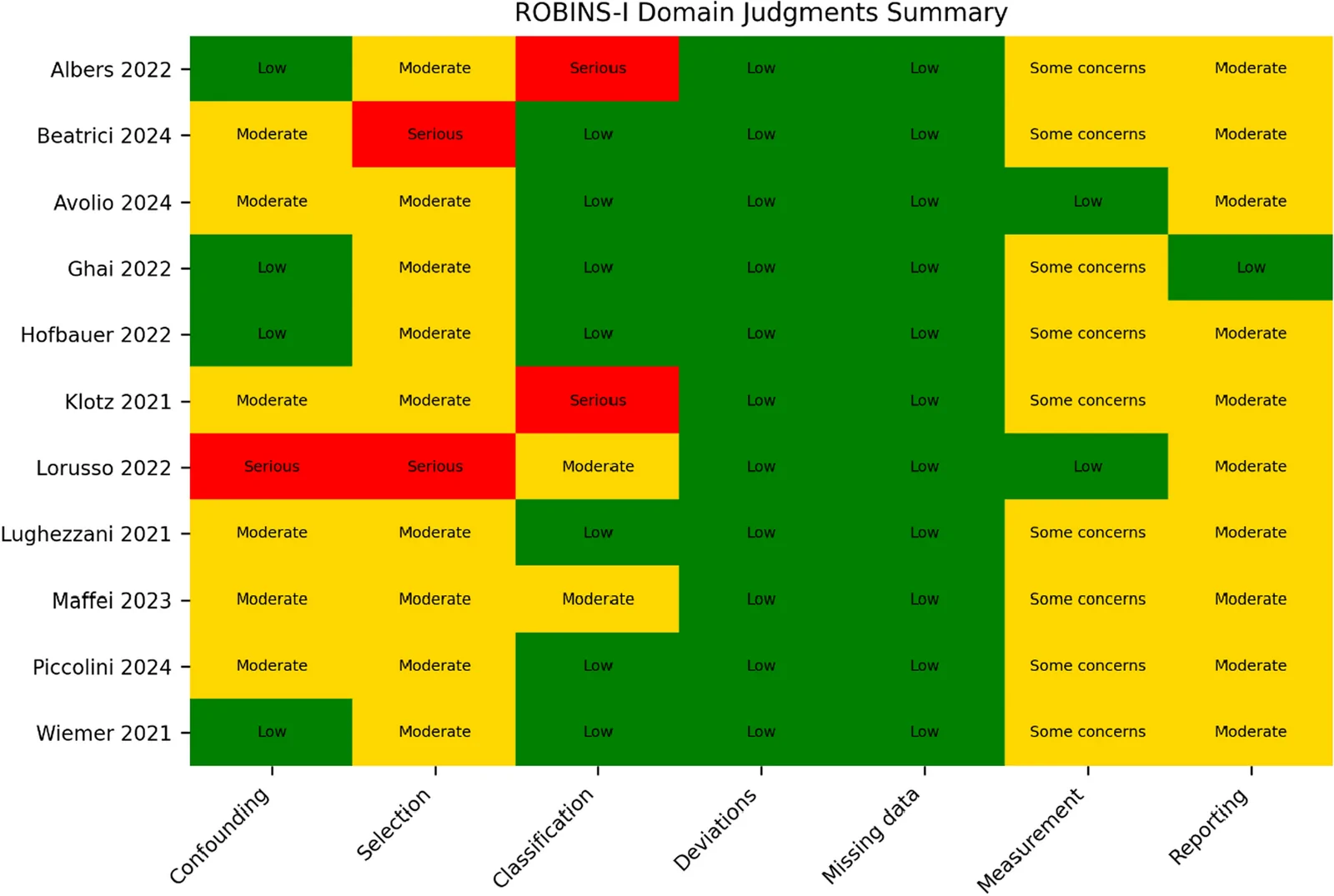
ROBINS-I I traffic-light summary across nonrandomized studies

Two reviewers independently extracted study characteristics and assigned judgments; disagreements were resolved by consensus. Where available, we used details from trial registration and methods sections to support judgments.

## Results

### Literature search

**Fig. 1:**
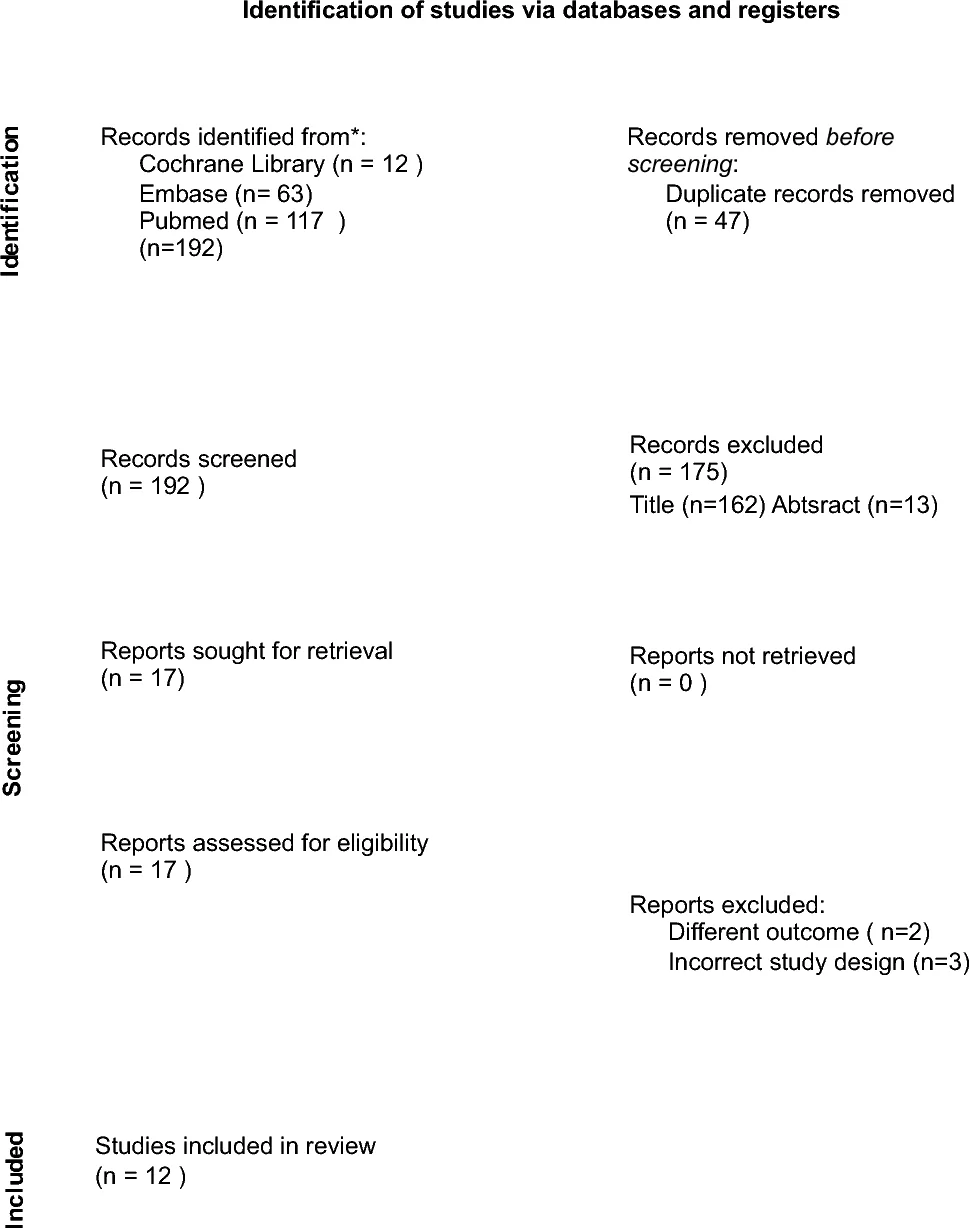
PRIMSA flowchart

After screening titles, abstracts, and full texts, 12 studies were deemed to meet the inclusion criteria. [6, 8–14, 17–20] The PRISMA flow diagram of study identification is outlined in Fig. 1. Three of the studies did not have a comparator group to allow inclusion in the meta-analysis but are discussed in the results of our systematic review. [9, 10, 13].

### Study characteristics

The studies were published between 2021 and 2025 and included 4318 patients, of whom 3475 had mUS-guided biopsies and 3,615 had mpMRI-guided biopsies. There were one RCT and eleven cohort studies. [6, 8–14, 17–20] The studies were published in Europe and North America. The largest study consisted of 1,094 patients, of whom 582 had mUS-guided biopsy and 512 had mpMRI-guided biopsy.

### Patient characteristics

The pooled mean age of all patients was 65 years. The range of mean ages was 63.7 to 69.3 years. There were 1,065 patients who had a suspicious digital rectal examination and the prevalence ranged from 14 to 100% across the studies included. The mean PSA was 7.2 ± 3.84 ng/mL and the range of mean PSA values was 6.5 to 8.6 ng/mL. The mean prostate volume was 50 ml and the mean volume ranged from 38.7 to 61.3 mL. Patient characteristics are outlined in Table 4.

**Table 4:** Patient demographics—studies included in meta-analysis

Author		Number (*n*)	Age (years) Mean (SD)	Suspicious DRE *n* (%)	Prostate volume (mL) Mean (SD)	PSA (µg/L) Mean (SD)
Avolio		182	N/A	182/182 (100%)	N/A	N/A
Beatrici		304	66.0 (7.45)	58/264 (22.0%)	59.7 (20.5)	8.6 (4.3)
Ghai		94	62.3 (9.0)	37/94 (39.4%)	49.0 (22.6)	6.6 (2.5)
Hofbauer		203	65.0 (8.2)	31/203 (15.3%)	38.7 (15.7) (Ultrasound)	6.9 (3.4)
					49.3 (19.4) (MRI)	
Kinnaird	mUS	121	65.3 (8.25)	25/121 (20.7%)	N/A	6.4 (4.1)
	mUS and MRI	226	64.17 (7.46)	48/226 (21.2%)		7.2 (3.1)
	MRI	331	65.3 (8.9)	81/331 (24.4%)		7.1 (3.3)
Klotz		1040	66.67 (8.17)	208/1040 (20.0%)	39.7 (18.6)	7.37 (3.6)
Maffei		100	65.0 (8.27)	14/100 (14%)	51.0 (22.6)	7.1 (3.4)
Piccolini	Micro US	582	64.3 (9.7)	143/582 (24.6%)	52.6 (23.9)	6.9 (3.0)
	MRI	512	64.7 (8.2)	140/512 (27.3%)	54.3 (24.5)	
Wiemer		159	69.3 (7.5)	42/159 (26.4%)	55.0 (30.7)	8.3 (4.3)
Studies included in narrative synthesis only						
Albers		128	65.8 (6.1)	19/128 (14.8%)	56.7 (27.7)	7.9 (4.0)
Lorusso		16	63.7 (16.3)	7/16 (43.8%)	61.3 (51.2)	8.2 (10.9)
Lughezzani		320	64.7 (8.19)	72/320 (22.5%)	48.3 (29.8)	7.5 (3.5)

*mUS* microultrasound, *MRI* magnetic resonance imaging, *DRE* digital rectal examination, *PSA* prostate specific antigen

### Primary outcome

Nine studies reported identification of PRIMUS lesions. PRIMUS 1 or 2 lesions were identified in 489/2,783 (17.6%) cases whilst PRIMUS 3–5 lesions were detected in 2,258/2,783 (81.1%) cases. Seven studies reported PIRADS 1 or 2 lesions whilst eight reported 3 to 5 lesions. PIRADS 1 or 2 lesions were present in 405/1,451 (27.9%) and PIRADS 3–5 lesions were present in 1,329/1,771 (75.0%) cases (Table 5).

**Table 5:** mUS and MRI findings

Author	Modality	Number (*n*)	PIRADS 1 Or PRIMUS 1	PIRADS 2 Or PRIMUS 2	PIRADS 3 Or PRIMUS 3	PIRADS 4 Or PRIMUS 4	PIRADS 5 Or PRIMUS 5	P1/2	P3-5
Albers	mUS	128	31/128 (24.2%)		36/128 (28.1%)	37/128 (28.9%)	24/128 (18.8%)	31/128 (24.2%)	97/128 (75.8%)
	MRI		55/128 (43.0%)		2/128 (1.6%)	39/128 (30.5%)	32/128 (25.0%)	55/128 (43.0%)	73/128 (57.0%)
Avolio	mUS	182	N/A	N/A	N/A	N/A	86/182 (47.3%)	22/182 (12.1%)	160/182 (87.9%)
Beatrici	mUS	304	N/A	N/A	27/304 (8.9%)	133/304 (43.8%)	60/304 (19.7%)	84/304 (27.6%)	220/304 (72.4%)
	MRI		N/A	N/A	57/304 (18.8%)	104/304 (34.2%)	40/304 (13.2%)	103/304 (33.9%)	201/304 (66.1%)
Hofbauer	mUS	203	9/203 (4.4%)	7/203 (3%)	56/203 (27.6%)	96/203 (47%)	35/203 (17%)	16/203 (7.9%)	187/203 (92.1%)
	MRI		1/203 (0.5%)	14/203 (7%)	37/203 (18.2%)	109/203 (54%)	41/203 (20%)	15/203 (7.4%)	187/203 (92.1%)
Kinnaird	mUS	121	4/121 (3.3%)	20/121 (16.5%)	21/121 (16.5%)	43/121 (35.5%)	24/121 (19.8%)	24/121 (19.8%)	88/121 (72.7%)
	mUS and MRI	226	5/226 (2.2%) 3/226 (1.3%)	42/226 (18.6%) 65/226 (32%)	38/226 (16.8%) 17/226 (7.5%)	68/226 (30.1%) 67/226 (33%)	57/226 (25.2%) 54/226 (26%)	47/226 (20.8%) 106/226 (46.9%)	163/226 (72.1%) 138/226 (61.1%)
	MRI	331	6/331 (1.8%)	105(34%)	42/331 (12.7%)	84(27%)	70(23%)	111/331	196/331
Klotz	mUS	1040	166/1040 (16.0%)		177/1040 (17.0%)	686/1040 (66.0%)		166/1040 (16.0%)	863/1040 (82.9%)
	MRI		N/A	N/A	198/1040 (19.0%)	N/A	N/A	N/A	N/A
Lughezzani	mUS	320	65/320 (20.3%)		38/320 (11.9%)	159/320 (49.7%)	58/320 (18.1%)	65/320 (20.3%)	255/320 (79.7%)
	MRI		N/A	N/A	56/320 (17.5%)	209/320 (65.3%)	55/320 (17.2%)	N/A	320/320 (100%)
Maffei	mUS	100	N/A	N/A	20/100 (20%)	50/100 (50%)	12/100 (12%)	18/100 (18%)	82/100 (82%)
	MRI		8/100 (8%)		28/100 (28%)	16/100 (16%)	18/100 (18%)	8/100 (8%)	62/100 (62%)
Wiemer	mUS	159	13/159 (8.2%)	3/159 (1.9%)	22/159 (13.9%)	74/159 (46.5%)	47/159 (29.5%)	16/159 (10.0%)	143/159 (89.9%)
	MRI		3/159 (1.9%	4/159 (2.5%)	15/159 (9.4%)	74/159 (46.5%)	63/159 (39.6%)	7/159 (4.4%)	152/159 (95.6%)

*mUS* micro-ultrasound, *MRI* magnetic resonance imaging, *PIRADS* Prostate Imaging Reporting and Data System, *PRIMUS* Prostate Risk Identification using micro-ultrasound, *N/A* not available

Ten studies reported prior biopsy experience with 2,193/3,624 (60.5%) biopsy-naive patients while 1,439/3,624 (39.7%) had a repeat biopsy. Seven studies reported biopsies as part of an active surveillance protocol which occurred in 379/1,663 (22.8%) patients (Table 6). Biopsies performed using MUS had a pooled mean of 4.5 targeted cores and 12.5 systematic cores while MRI-guided biopsies had a pooled mean of 3.3 targeted cores and 12.2 systematic cores (Table 7).

**Table 6:** Nature of biopsy

Author	Number (n)	First biopsy	Re-biopsy	Active surveillance
Albers	128	0/128 (0%)	128/128 (100%)	128/128 (100%)
Avolio	182	182/182 (100%)	0/182 (0%)	0/182 (0%)
Beatrici	304	0/304 (0%)	304/304 (100%)	0/0 (0.0%)
Ghai	94	94/94 (100%)	0/94 (0%)	N/A
Hofbauer	203	147/203 (72%)	56/203 (27.5%)	N/A
Klotz	1040	688/1040 (66.1%)	352/1040 (33.8%)	N/A
Lughezzani	320	200/320 (62.5%)	120/320 (37.5%)	N/A
Maffei	100	0/100 (0%)	100/100 (100%)	100/100 (100%)
Piccolini				
mUS	582	418/582 (71.8%)	164/582 (28.2%)	46/582 (7.9%)
MRI	512	385/512 (75.2%)	127/512 (24.8%)	59/512 (11.5%)
Wiemer 2021	159	79/159 (49.7%)	88/159 (55.3%)	46/159 (28.9%)

*mUS* microultrasound, *MRI* magnetic resonance imaging, *N/A* not available

**Table 7:** Biopsy cores

Author	Modality	Number (n)	Targeted Cores Mean (SD)	Systematic Cores Mean (SD)
Albers	mUS	128	3	12
	MRI		3	12
Beatrici	mUS	304	N/A	N/A
	MRI		N/A	> 6
Ghai	mUS	94	N/A	N/A
	MRI		2	12
Lughezzani	mUS	320	N/A	N/A
	MRI		4.3 (2.2)	12.7 (3.7)
Piccolini	mUS	582	4.3 (2.2)	14 (2.9)
	MRI	512	3 (1.5)	13.7 (2.2)
Wiemer	mUS	159	5.7 (5.2)	6.33 (2.2)
	MRI		2.0 (0)	6.33 (2.2)

*mUS* microultrasound, *MRI* magnetic resonance imaging, *N/A* not available

**Fig. 2:**
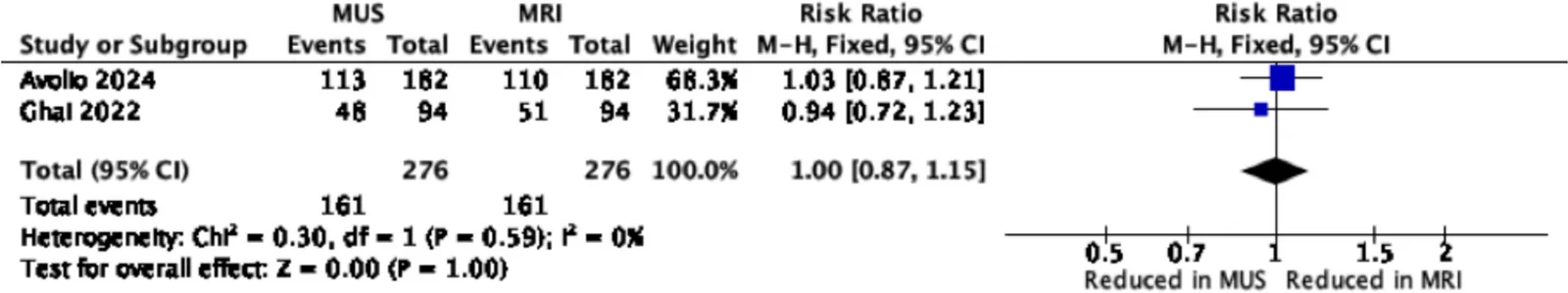
Pooled proportions of overall cancer detection in mUS-guided and MRI-guided biopsies. *CI*, confidence interval

**Fig. 3:**
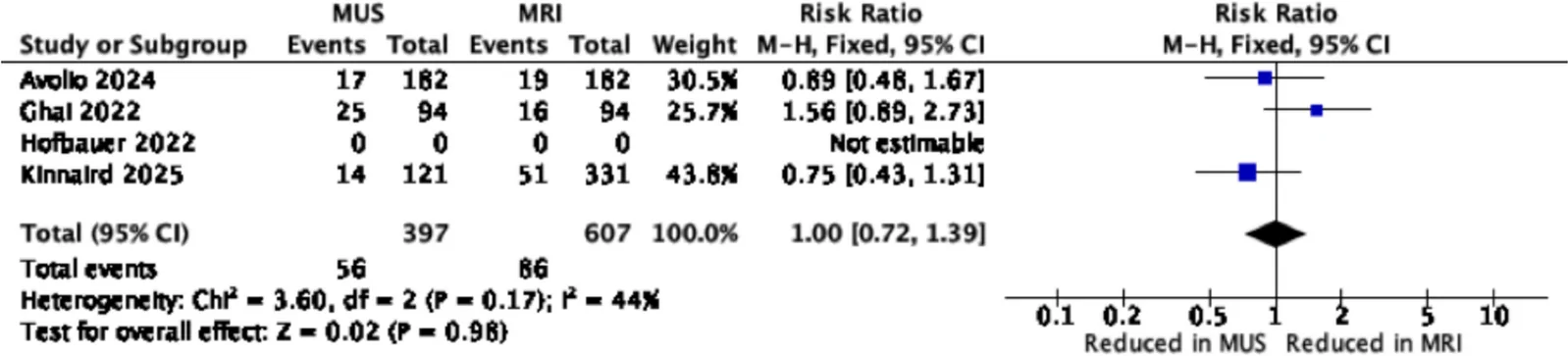
Pooled proportions of overall ISUP GG1 cancer detection in mUS-guided and MRI-guided biopsies. *CI* confidence interval

**Fig. 4:**
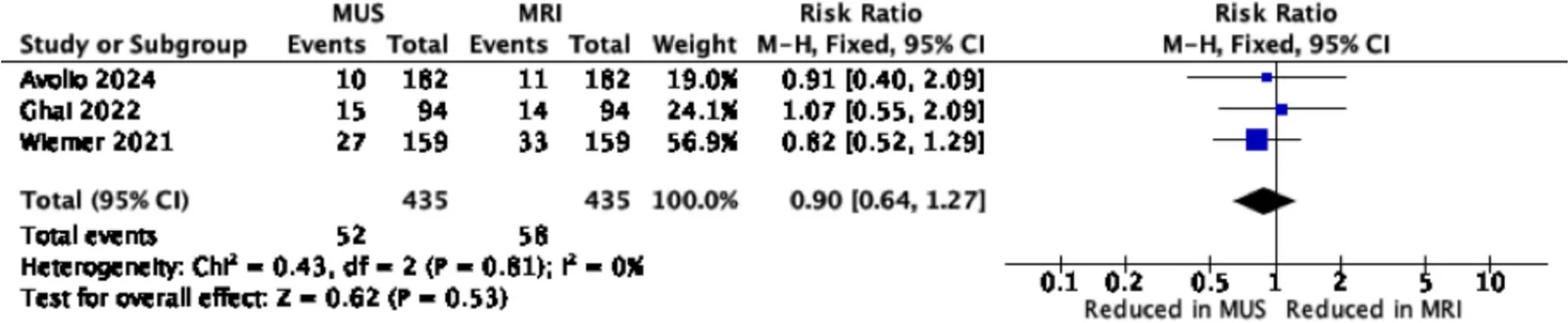
Pooled proportions of overall ISUP GG1 cancer detection in targeted biopsies in mUS-guided and MRI-guided biopsies, *CI* confidence interval

**Fig. 5:**
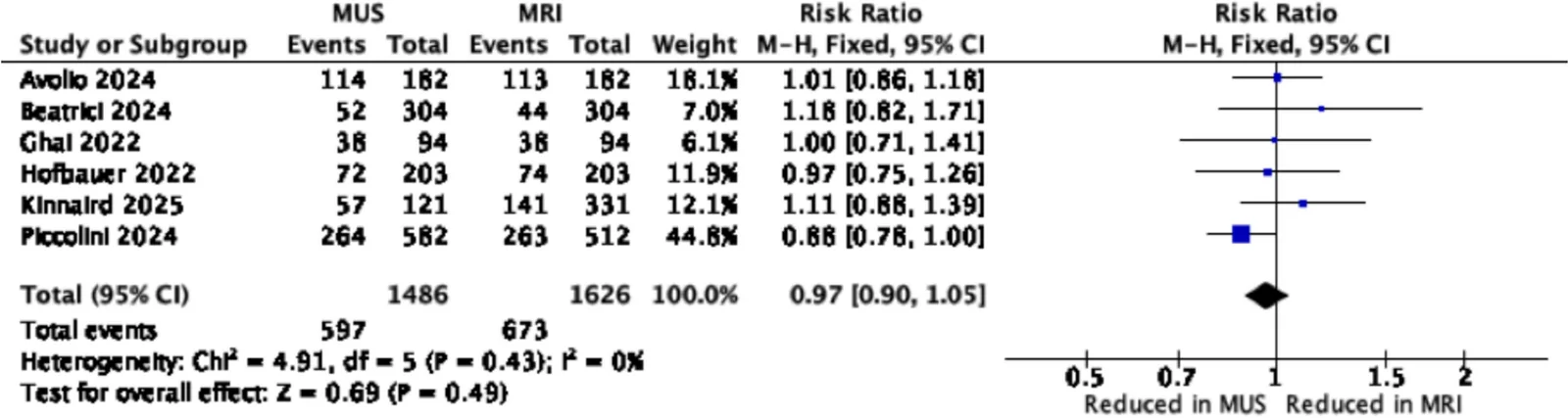
Pooled proportions of overall csPCa cancer detection in mUS and MRI biopsies, *CI* confidence interval

**Fig. 6:**
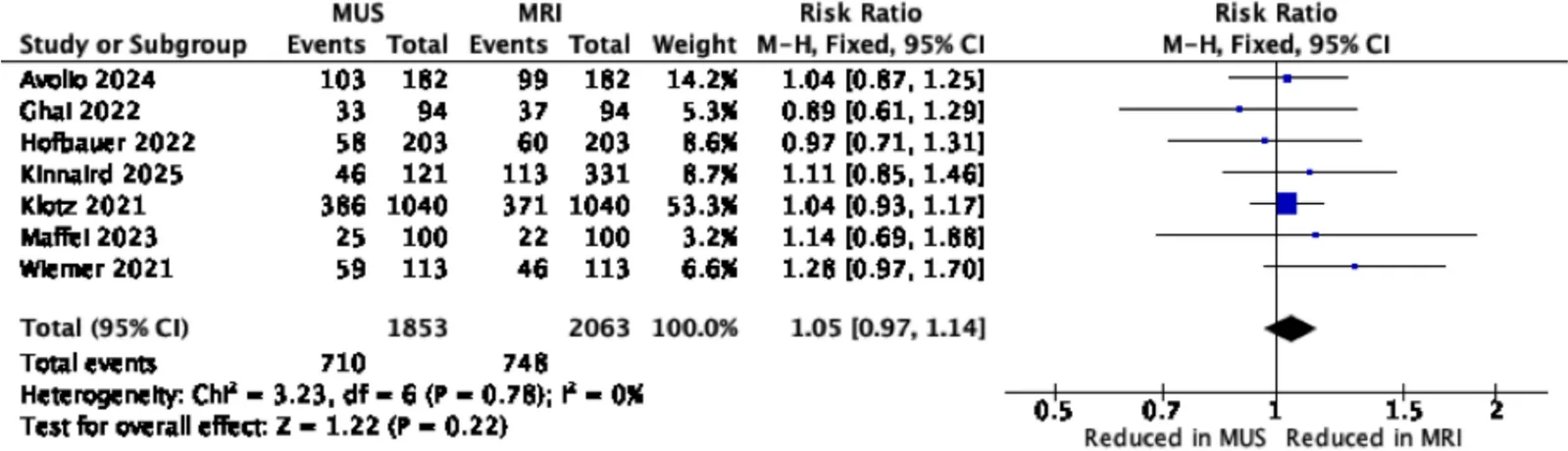
Pooled proportions of csPCa detection in targeted biopsies in mUS-guided and MRI-guided biopsies, *CI* confidence interval

All studies reported cancer detection rates. The overall cancer detection rate was reported in 11 studies and was 60.0% of patients (2,045/3,406). There was no significant difference between mUS-guided and MRI-guided biopsy in overall prostate cancer detection (RR 1.00, 95% CI 0.87 to 1.15, *p* = 0.59). Similarly, detection of Grade Group 1 cancer did not differ between groups, whether assessed across all biopsies (RR 1.00, 95% CI 0.72 to 1.39) or with targeted biopsy alone (RR 0.90, 95% CI 0.64 to 1.27). The rate of clinically significant prostate cancer detection was likewise comparable, both overall (RR 0.97, 95% CI 0.90 to 1.05) and with targeted biopsy (RR 1.05, 95%CI 0.97 to 1.14) (Table 8 and Figs. 2, 3, 4, 5 and 6).

**Table 8:** Cancer detection

Author/Modality		Overall cancer detection	Overall cancer detection targeted biopsies (*n*)	Overall GG1 (*n*)	GG1 targeted biopsy (*n*)	Overall csPCa (*n*)	csPCa targeted biopsy (*n*)
Albers		100/128 (78.1%)	N/A	66	N/A	34	N/A
Avolio	mUS	131/182 (72.0%)	113	17	10	114	103
	MRI	132/182 (72.5%)	110	19	11	113	99
Beatrici	mUS	102/304 (33.6%)	N/A	45	N/A	52	N/A
	MRI					44	
Ghai	mUS	63/94 (67.0%)	48	25	15	38	33
	MRI		51	16	14		37
Hofbauer	mUS	127/203 (62.6%)	N/A	48	N/A	72	58
	MRI					74	60
Kinnaird	mUS	71/121 (58.7%)	N/A	14	N/A	57	46
	mUS + MRI	145/226 (64.2%)		39		106	91
	MRI	192/331 (58.0%)		51		141	113
Klotz	mUS	632/1040 (61%)	N/A	N/A	N/A	411	386
	MRI						371
Lughezzani		156/320 (48.7%)	N/A	40	N/A	116	N/A
Lorusso		16/16 (100%)	N/A	N/A	N/A	N/A	N/A
Maffei	mUS	65/100 (65%)	N/A	31	N/A	34	25
	MRI						22
Piccolini	mUS	N/A	N/A	N/A	N/A	264	N/A
	MRI	N/A				263	
Wiemer	mUS	113/159 (71.1%)	59	N/A	27	78	59
	MRI		46		33		46

*mUS* microultrasound, *MRI* magnetic resonance imaging, *csPCa* clinically significant prostate cancer, *N/A* not available

For csPCa, MUS had a pooled sensitivity of 93.2%, specificity of 23.3%, positive predictive value (PPV) of 46.4% and negative predictive value (NPV) of 81.2%. MRI had a pooled sensitivity of 87.5%, specificity of 25.7%, PPV of 44.6% and NPV of 73.7%.

## Discussion

In this systematic review and meta-analysis of 12 comparative studies, we found no significant difference between micro-ultrasound (mUS)-guided and multiparametric MRI (mpMRI)-guided biopsy for detecting clinically significant prostate cancer (csPCa), both overall (RR 0.97; 95%CI 0.90–1.05) and with targeted biopsy (RR 1.05; 95%CI 0.97–1.14). Overall cancer detection and Grade Group 1 detection were likewise comparable. Pooled test characteristics favoured mUS for sensitivity and NPV, with broadly similar specificity and PPV compared with mpMRI.

Our results are concordant with the OPTIMUM randomized clinical trial (RCT), which demonstrated noninferiority of microultrasonography-guided targeted biopsy versus MRI/US fusion-guided biopsy for detection of Grade Group ≥ 2 cancer in biopsy-naïve men. In OPTIMUM, the absolute difference for csPCa detection was 3.52% (95%CI − 3.95% to 10.92%), meeting the prespecified noninferiority margin (*p* < 0.001) [8].

This RCT context strengthens our meta-analytic inference in several ways. First, OPTIMUM enrolled biopsy-naïve patients and compared real-time mUS targeting with contemporary MRI/US fusion, directly addressing whether MRI is required for initial image-guided biopsy. Second, the noninferiority effect size in OPTIMUM mirrors our pooled estimate showing diagnostic equivalence of modalities for csPCa in these selected cohorts. Third, post-publication commentary highlighted potential economic and environmental advantages of an ultrasound-based strategy when diagnostic yield is noninferior—considerations that amplify the health-system relevance of our findings.

Although OPTIMUM provides high-level evidence, our synthesis broadens generalisability by incorporating biopsy-naïve, prior-negative and active surveillance cohorts along with both transperineal and transrectal approaches. Heterogeneity in operator experience, targeting schema, and reference standards persists across the literature, but the directionality is stable: mUS performs on par with MRI-guided biopsy for csPCa detection in the included studies [6–8].

Taken together, our results and OPTIMUM suggest that centres with mUS expertise can reasonably offer an mUS-first pathway for men with suspected prostate cancer—particularly where access to high-quality MRI is constrained, or where single-visit diagnosis is desirable. This does not displace MRI’s roles in local staging, assessment of extra prostatic extension, treatment planning, and evaluation of challenging anatomic regions; rather, it supports tailoring the sequence of imaging based on local resources and patient factors [6, 13]. Current EAU/AUA guidance still centres MRI in prebiopsy pathways, but emerging evidence indicates mUS is a credible alternative or complement to MRI/fusion targeting pending integration into standard prostate cancer diagnostic guidelines.

## Strengths and limitations

Strengths of our study include a comprehensive search to April 2025, inclusion of the contemporary literature, and pooled estimates across diverse clinical contexts. This review assessed several clinically relevant endpoints, including overall prostate cancer detection, Grade Group 1 cancer detection, clinically significant prostate cancer detection, targeted biopsy outcomes, and pooled diagnostic performance metrics including sensitivity, specificity, PPV, and NPV. The inclusion of studies across different clinical contexts, including biopsy-naïve men, patients undergoing repeat biopsy, and men on active surveillance, provides a broad overview of how mUS performs across real-world diagnostic settings. Finally, by incorporating the recent OPTIMUM randomized clinical trial [8] alongside observational data, this review contextualises emerging high-level evidence within the wider clinical literature and provides a contemporary summary of the potential role of mUS as an alternative or adjunct to mpMRI-guided biopsy.

Several limitations should be acknowledged. There was important clinical heterogeneity across the included studies. Patient populations differed with respect to biopsy history, indication for biopsy, and baseline risk. Some studies enrolled biopsy-naïve men, whereas others included patients undergoing repeat biopsy after prior negative biopsy or men on active surveillance protocols. These groups may differ in cancer prevalence, lesion conspicuity, prior sampling, and pre-test probability of clinically significant disease. As a result, pooled detection estimates should be interpreted as reflecting an aggregate across mixed clinical settings rather than a single uniform diagnostic pathway, which reflects real world practice.

Biopsy technique varied across studies, as did whether mUS or mpMRI was used as a standalone targeting modality or as part of a combined diagnostic strategy. The inclusion of systematic cores may dilute the independent contribution of each imaging modality to cancer detection.

Efforts were made to include the most relevant dataset where duplicate populations were apparent. The possibility of residual overlap cannot be excluded, reducing generalisability.

Most included studies were observational. Although the OPTIMUM trial provides high-quality evidence supporting non-inferiority of mUS-guided biopsy in biopsy-naïve men, the broader evidence base remains subject to centre-level expertise effects. This is particularly relevant because mUS performance may be influenced by the learning curve associated with image acquisition, PRI-MUS interpretation, and real-time targeting.

## Conclusion

mUS-guided biopsy demonstrates diagnostic performance comparable to mpMRI-guided biopsy for PCa detection and represents a promising alternative or adjunct to mpMRI in prostate cancer diagnostic pathways in select centres with expertise in the use of this operator dependent imaging technique., Given heterogeneity in patient populations, biopsy pathways, scoring systems, and study design, these findings should be interpreted cautiously and validated in further multicentre randomized studies using standardized biopsy protocols and robust reference standards. ideally using whole-mount prostatectomy specimens as the definitive reference standard to allow adoption of this technique into our current clinical guidelines for prostate cancer diagnostics. Formal cost effectiveness analysis should be performed to lend weight to the wider scale adoption of this technique.

## Data Availability

All data supporting the findings of this study are available within the paper and its references.

## Abbreviations

TRUS: Transrectal ultrasound
mpMRI: Multiparametric magnetic resonance imaging
csPCa: Clinically significant prostate cancer
mUS: Microultrasound
